# A Novel Benzothiazole-Based Fluorescent AIE Probe for the Detection of Hydrogen Peroxide in Living Cells

**DOI:** 10.3390/molecules29215181

**Published:** 2024-11-01

**Authors:** Dezhi Shi, Yulong Yang, Luan Tong, Likang Zhang, Fengqing Yang, Jiali Tao, Mingxia Zhao

**Affiliations:** 1 The Cultivation Base of Shanxi Key Laboratory of Mining Area Ecological Restoration and Solid Wastes Utilization, Shanxi Institute of Technology, Yangquan 045000, China; shidezhi_sxit@163.com (D.S.); yangfengqingq@163.com (F.Y.); 18306891943@163.com (J.T.); 2 Yangquan Technology Innovation Center of Carbon Dioxide Capture, Utilization and Storage, Shanxi Institute of Technology, Yangquan 045000, China; 3 Department of Veterinary Medicine, Shanxi Agricultural University, Jinzhong 030801, China; buzzlightyang@163.com (Y.Y.); tongluan0101@163.com (L.T.); 15835459393@163.com (L.Z.)

**Keywords:** fluorescence probe, AIE probe, hydrogen peroxide, benzothiazole base derivative

## Abstract

A benzothiazole-based derivative aggregation-induced emission (AIE) fluorescent ‘turn-on’ probe named 2-(2-((4-(4,4,5,5-tetramethyl-1,3,2-dioxaborolan-2-yl)benzyl)oxy)phenyl)benzo[*d*]thiazole (probe **BT-BO**) was developed and synthesized successfully for detecting hydrogen peroxide (H_2_O_2_) in living cells. The synthesis method of probe **BT-BO** is facile. Probe **BT-BO** demonstrates a well-resolved emission peak at 604 nm and the ability to prevent the interference of reactive oxygen species (ROS), various metal ions and anion ions, and good sensitivity. Additionally, the probe boasts impressive pH range versatility, a fast response time to H_2_O_2_ and low cytotoxicity. Finally, probe **BT-BO** was applied successfully to image A549 and Hep G2 cells to monitor both exogenous and endogenous H_2_O_2_.

## 1. Introduction

Reactive Oxygen Species (ROS) are highly reactive oxygen-containing compounds that play an important role in living cells [1,2]. Hydrogen peroxide (H_2_O_2_), one of the typical representatives of ROS, maintains a balance between oxidative and antioxidant systems in normal cells [3,4]. Nonetheless, once this balance is broken, for example, if the concentration of H_2_O_2_ is raised or the antioxidant capacity decreases, damage is caused to living cells, resulting in the destruction of nucleic acids, proteins and enzymes, and diseases [3,5,6,7,8]. During an inflammatory process, cells become activated and several mediators, including tissue factors, are released. In inflammatory responses, ROS can aggravate local tissue damage and lead to chronic inflammation [9]. In cancer therapy, H_2_O_2_ is a double-edged sword. On the one hand, the H_2_O_2_ level in tumor cells is higher than that in normal cells, which causes them to be in a state of oxidative stress, and provides a theoretical basis for some pro-oxidation drugs to treat cancer [10]. On the other hand, the excessive production and accumulation of H_2_O_2_ in cells can result in various diseases such as cancer, arthrophlogosis, asthma and cardiovascular diseases [11,12,13].

There are numerous methods for detecting H_2_O_2_, such as electron paramagnetic resonance (EPR)/electron spin resonance (ESR) techniques [14], chemiluminescence [15], th electrochemical method [16] and chromatography [17]. However, these methods exhibit limitations in applications, high costs and so on [18]. Currently, fluorescence spectroscopy has drawn the increasing attention of researchers and has been widely used in the field of detecting H_2_O_2_ in living cells, due to its high selectivity, high sensitivity, high efficiency, simple operation, good biocompatibility, wide range of application and real-time imaging [19,20,21,22,23]. Nevertheless, conventional organic fluorescent probe molecules have aggregation-caused quenching (ACQ) effects, i.e., fluorescence is partially or completely quenched [3,12,24,25,26]. The ACQ phenomenon results in low fluorescence signals, poor sensitivity, and easy photobleaching in biological detection [27]. In 2001, for the first time, Prof. Tang’s group found the aggregation-induced emission (AIE) effect of 1-methyl-1,2,3,4,5-pentaphenylsilole, which was ascribed to the limited rotations of rotatable meso moieties in closed spaces [28,29]. The principle of AIE is based on the restriction of intramolecular motion (RIM) or the restriction of intramolecular rotation (RIR). Molecules with AIE properties exhibit weak or even unobservable fluorescence features in dilute solutions, but they can fluoresce brightly when aggregated in a solution state [30], which effectively avoids the ACQ problem of traditional fluorescent molecules. Since 2001, a series of well-designed fluorescent probes that can specifically detect H_2_O_2_ have been developed. Tang et al. [18] explored a benzothiazole-based fluorescence probe for detecting hydrogen peroxide, which exhibited good sensitivity, selectivity and low cytotoxicity in HeLa cells. Liu et al. [26] developed an archetypal AIE luminogen (tetraphenylethene-based) H_2_O_2_ fluorescent probe, which shows high sensitivity, high selectivity, low cytotoxicity, and good cell membrane permeability. Zhong et al. [25] developed a new type of “turn-on” AIE probe based on excited-state intramolecular proton transfer (ESIPT), with phenylboronic acid pinacol ester-appended quinolinium used as the H_2_O_2_ recognition site due to the twisted intramolecular charge transfer (TICT) effect. Currently, boric acid or boric acid ester are widely used as H_2_O_2_-responsive elements [18,31,32]. ESIPT represents a distinctive four-stage photochemical process, characterized by fluorophores that predominantly reside in the electronic ground state, often manifesting as enols [18]. Upon light excitation, the charge distribution within this type of molecule is altered, leading to the enhanced acidity of the hydrogen bond donor in its enol form and a corresponding increase in the basicity of the hydrogen bond acceptor. Ultimately, phototautomerization leads to the excited enol form quickly converting to an excited keto structure. Upon returning to the ground state, reverse proton transfer (RPT) restores the original enol form [33]. 2-(2’-hydroxyphenyl)benzothiazole (HBT) is a common ESIPT fluorophore and its derivative has been widely used as a ESIPT fluorescent probe for detecting H_2_O_2_ [33,34].

Based on the AIE-ESIPT principle, in this work, an aryl boric acid ester unit was introduced into a benzothiazole-based derivative fluorophore (HBT) to serve as the reactive group for H_2_O_2_. The new ‘turn on’ fluorescent probe, 2-(2-((4-(4,4,5,5-tetramethyl-1,3,2-dioxaborolan-2-yl)benzyl)oxy)phenyl)benzo[*d*]thiazole (denoted as probe **BT-BO**), was designed and synthesized successfully for the first time for the selective detection of H_2_O_2_. Probe **BT-BO** exhibited a well-resolved emission peak at 604 nm and the ability to prevent the interference of ROS, various metal ions and anion ions. Moreover, the probe possessed the excellent properties of a broad pH range, a fast response time to H_2_O_2_ and low cytotoxicity. Finally, we have successfully applied the probe **BT-BO** to image A549 and Hep G2 cells to monitor both exogenous and endogenous H_2_O_2_.

## 2. Results and Discussion

### 2.1. Synthesis and Structural Analysis of HBT and Probe BT-BO

**Figure 1:**
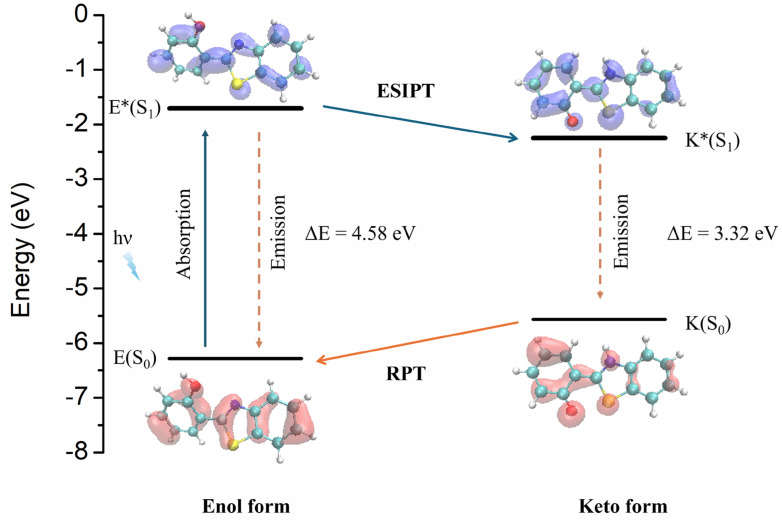
Energy gaps and diagrammatic description of the ESIPT process in this work. (E*: excited enol form, E: enol form; K* excited keto form, K: keto form).

**Scheme 1:**
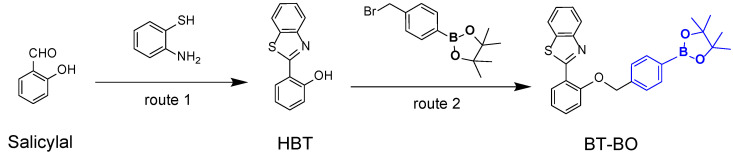
Route 1: Anhydrous ethanol as a solution with two drops of formic acid under refluxing for 2.5 h, with a yield of 76.5%; route 2: anhydrous acetonitrile as a solution with potassium carbonate under refluxing for 4 h, with a yield of 76.4%.

**Scheme 2:**
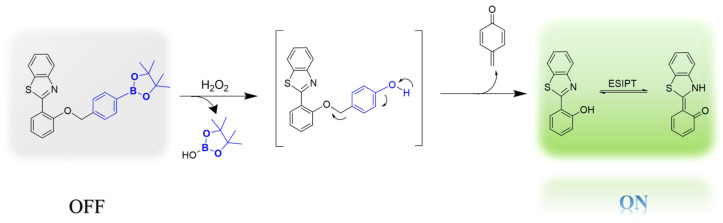
Proposed response mechanism of “turn-on” fluorescent probe **BT-BO** designed to monitor H_2_O_2_.

The synthesis route of the fluorescent probe **BT-BO** used to monitor H_2_O_2_ is illustrated in Scheme 1. Probe **BT-BO** was synthesized via two simple routes: in step 1, salicylal (2-hydroxybenzaldehyde) was refluxed with 2-aminobenzenethiol in a anhydrous ethanol solution to obtain HBT (2-(2’-hydroxyphenyl)benzothiazole); in step 2, the arylboronate ester group was chosen as the recognition moiety to detect H_2_O_2_, which was refluxed with HBT to produce probe **BT-BO** (2-(2-((4-(4,4,5,5-tetramethyl-1,3,2-dioxaborolan-2-yl)benzyl)oxy)phenyl)benzo[*d*]thiazole) under a solution of anhydrous acetonitrile. The rational design of probe **BT-BO** is based on using the arylboronate ester group as a H_2_O_2_-responsive unit, which shows a “turn-on” fluorescent signal characteristic that recognizes and responds to H_2_O_2_ [29] (Scheme 2, Figure S1). Moreover, The ESIPT mechanism of HBT was verified by DFT calculations. Figure 1 shows a four-stage photochemical ESIPT process concerning the phototautomerization of the excited enol form to the excited keto form. The energy difference between the LUMO orbital and the HOMO orbital of the keto form (3.32 eV) is smaller than that of the enol form (4.58 eV), which confirmed the ESIPT process of HBT.

The structures of HBT and probe **BT-BO** were characterized by ^1^H NMR, ^13^C NMR and MS (Figures S2–S7 in Supplementary Materials). The HR-MS spectra of HBT exhibit an intense ion peak at *m*/*z* = 228.0487 (M + H), which is ascribed to its corresponding ion [**HBT** + H]^+^, and match up well with the calculated *m*/*z* of 228.0483 of [M + H]^+^ (Figure S6). The same result was found for probe **BT-BO**, with an ion peak at an *m*/*z* value of 444.1801 for [**BT-BO** + H]^+^ and a calculated *m*/*z* value of 444.1805 (Figure S7). Moreover, the ^1^H NMR and ^13^C NMR results also confirmed the structures of HBT and probe **BT-BO;** details can be found in Section 3.2 and Figures S2–S5 and Tables S2–S3 of the Supplementary Materials.

To further verify the structure of probe **BT-BO**, we cultured a single crystal of **BT-BO**. A suitable crystal of **BT-BO** was grown by slowly evaporating the solution of THF at room temperature. Details of the crystallographic and structure refinement for **BT-BO** are given in Figure S8 and Table S1. The crystal structure and ^1^H NMR, ^13^C NMR, and HR-MS all confirm that probe **BT-BO** forms a benzothiazole structure.

### 2.2. AIE Property of HBT

**Figure 2:**
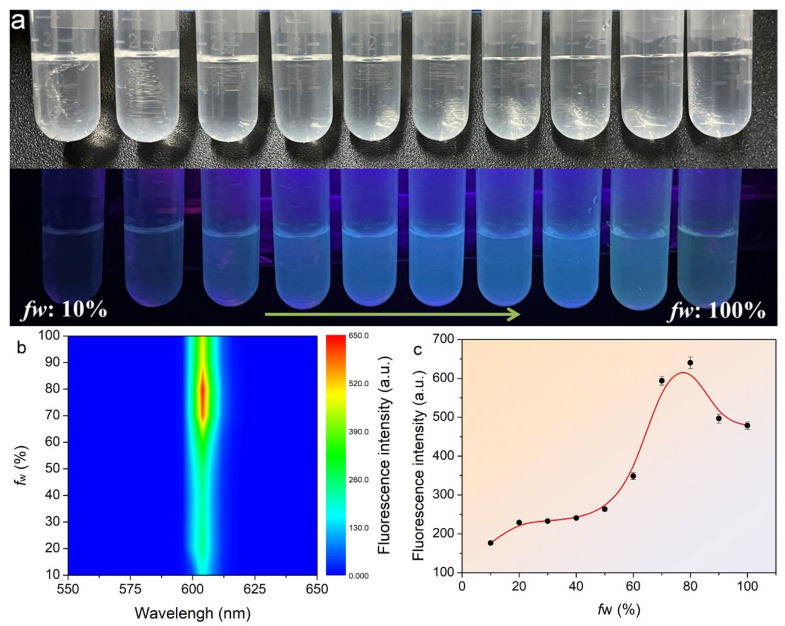
(**a**) Photographs of HBT in water/THF mixtures obtained from light and UV illumination; (**b**) Fluorescence spectra of HBT in water/THF mixtures; (**c**) Plots of maximum emission intensity of HBT with different *f*_w_ values. λ_ex_ = 312 nm.

To verify the AIE property of HBT, different water fractions (*f*_w_) in tetrahydrofuran (THF) solution were used to investigate the fluorescence behavior of HBT. As Figure 2a shows, photographs of HBT in water/THF mixtures were taken under visible light. However, when *f*_w_ was increased, a sapphire solution emerged; this then darkened slightly under ultraviolet light. In order to further quantify the phenomena, the emission spectra of HBT in different *f*_w_ in the THF solution were measured (Figure 2b). The fluorescence intensity at 604 nm gradually increased and then slightly lowered with an increase in the *f*_w_ value (Figure 2c). The above results indicate that HBT was a fluorescent material with AIE properties.

### 2.3. Spectral Properties of Probe BT-BO to H_2_O_2_

**Figure 3:**
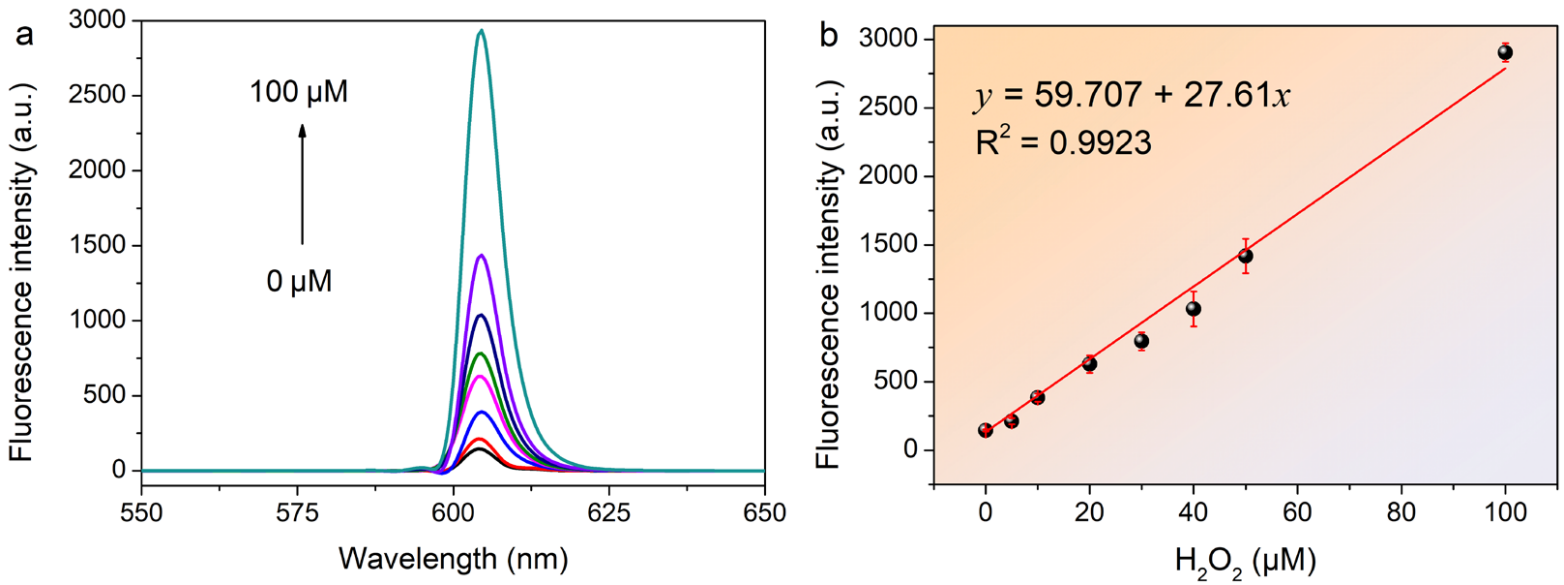
(**a**) Fluorescence spectra of probe **BT-BO** (5 μM) after the addition of different concentrations of H_2_O_2_ (0, 5, 10, 20, 30, 40, 50, 100 μM, respectively) λ_ex_ = 324 nm. (**b**) Linear correlation between the fluorescence intensity of probe **BT-BO** (5 μM) at 604 nm and H_2_O_2_ concentrations of 0, 5, 10, 20, 30, 40, 50 and 100 μM.

In order to investigate the fluorescence characteristics of probe **BT-BO**, probe **BT-BO** (5 μM) was treated with different concentrations of H_2_O_2_ in phosphate-buffered saline (PBS) solution (containing 2% THF, vol). We investigated the change in the fluorescence spectra signal of various concentrations of H_2_O_2_ (0~100 μM). As shown in Figure 3a, probe **BT-BO** exhibited weak fluorescence due to blocking from the aryl boric acid ester moiety, indicating the low fluorescence emission background of probe **BT-BO** itself. After adding various concentrations of H_2_O_2_, the fluorescence emission intensity at 604 nm was gradually increased, suggesting that H_2_O_2_ was indeed reacted with probe **BT-BO**. The fluorescence intensity at 604 nm for a H_2_O_2_ concentration of 100 μM was 20 times that for 0 μM of H_2_O_2_, which means that probe **BT-BO** has a high signal-to-noise ratio. The experimental results showed that the probe was a turn-on fluorescent probe, laying the foundation for its detection of H_2_O_2_ [33]. In addition, a relationship between the fluorescence intensity of probe **BT-BO** and the H_2_O_2_ concentration was fitted. The linear regression equation was *y* = 59.707 + 27.61*x* and the linear correlation coefficient R^2^ was 0.9923, where *y* was the fluorescence intensity of probe **BT-BO** at 604 nm; *x* refers to the H_2_O_2_ concentration. The limit of detection (LOD) for H_2_O_2_ was estimated based on the following rule [8,18,35,36,37,38,39,40,41,42,43,44,45,46]:

LOD = 3*σ*/*k*

where *σ* is the standard deviation of blank fluorescence intensity measurements, which were detected 11 times, and *k* refers to the slope of the fitting curve (Figure 3b). The LOD value of probe **BT-BO** was determined to be 0.93 μM (3 × 8.54/27.61 = 0.93 μM), suggesting that probe **BT-BO** could be a good “turn-on” response sensor for determining H_2_O_2_.

### 2.4. Selectivity, Effect of pH, Temperature and Response Time of Probe BT-BO

**Figure 4:**
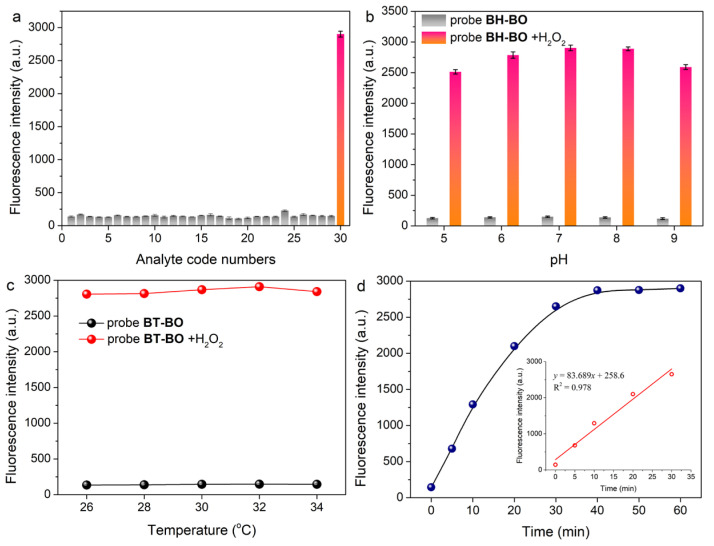
(**a**) Fluorescence responses of probe **BT-BO** (5 μM) at 604 nm in the presence of various relevant analytes: (1) N_3_^−^, (2) F^−^, (3) HPO_4_^2−^, (4) SO_4_^2−^, (5) HSO_4_^−^, (6) Cl^−^, (7) OH^−^, (8) CN^−^, (9) CO_3_^2−^, (10) HCO_3_^−^, (11) NO_3_^−^, (12) NO_2_^−^, (13) CH_3_COO^−^, (14) Ba^2+^, (15) Fe^2+^, (16) Fe^3+^, (17) Ca^2+^, (18) K^+^, (19) Al^3+^, (20) Mg^2+^, (21) Na^+^, (22) Cu^2+^, (23) GSH, (24) ONOO^–^, (25) TBHP, (26)·O_2_^−^, (27) HClO, (28) ^1^O_2_, (29) blank, (30) H_2_O_2_. The concentration of interfering analytes was 1000 μM, and the concentration of H_2_O_2_ was 100 μM. (**b**) Fluorescence emission of probe **BT-BO** (5 μM) at 604 nm with (pink) or without (light gray) H_2_O_2_ at different pH values. (**c**) Fluorescence intensity of probe **BT-BO** (5 μM) at 604 nm with (red) or without (black) H_2_O_2_ (100 μM) at different temperatures. (**d**) Time-dependent profile of probe **BT-BO** (5 μM) at 604 nm with H_2_O_2_ (100 μM) and linear fitting curve of time-dependent fluorescence intensity of probe **BT-BO** in the presence of H_2_O_2_. λ_ex_ = 324 nm.

The selectivity and anti-interference capabilities of probe **BT-BO** were investigated using blank and various reactive oxygen species (ROS), namely glutathione (GSH), peroxynitrite (ONOO^–^), tert-butyl hydroperoxide (TBHP), O_2_^−^, HClO, ^1^O_2_, and H_2_O_2_; various metal ions, namely Ba^2+^, Fe^2+^, Fe^3+^, Ca^2+^, K^+^, Al^3+^, Mg^2+^, Na^+^, Cu^2+^; and various anion ions, namely N_3_^−^, F^−^, HPO_4_^2−^, SO_4_^2−^, HSO_4_^−^, Cl^−^, OH^−^, CN^−^, CO_3_^2−^, HCO_3_^−^, NO_3_^−^, NO_2_^−^, CH_3_COO^−^ and blank. As illustrated in Figure 4a, the fluorescence intensity of probe **BT-BO** in the solution of H_2_O_2_ (100 μM) was significantly higher than that of potential interferences, even at a high concentration level (1000 μM). The response intensity of H_2_O_2_ was about 17~25 times that of other potential interferences. These results indicate that probe **BT-BO** has high selectivity for H_2_O_2_, which means that probe **BT-BO** could play an active role in endogenous H_2_O_2_ imaging in living cells.

For comparison, we have summarized some of the previously reported fluorescent probes used to compare the Limit of Detection (LOD), response time, selectivity, practical applications, and synthesis of **BT-BO** and previously reported fluorescent probes for detecting H_2_O_2_. Most of the LOD values obtained by previously reported fluorescent probes are around 0.13~5.3 μM, while our designed and synthesized probe possesses a LOD value of 0.93 μM; that is, **BT-BO** has comparability and high sensitivity for detecting H_2_O_2_ (Table S4). The comparison of the selectivity and anti-interference capabilities of the reported probes and **BT-BO** show that the lowest fluorescent response of H_2_O_2_ is around 2.26~13.75 better than interferences. However, this value for **BT-BO** is 17.02. The number of interference types investigated in the previously reported literature is 7~22, while the number in our work is 29. Moreover, we compare the synthesis steps and application cells between previous studies and this work. The number of synthesis steps and application cells used in the previously reported literature is 2~5 steps and 1~3 cells, respectively. Obviously, probe **BT-BO** also has comparability (two synthesis steps and two application cells). All in all, these preliminary experiments suggest that probe **BT-BO** could be a potential probe for detecting H_2_O_2_.

To investigate the effect of pH values on the experimental results, the fluorescence emission properties of probe **BT-BO** before and after adding H_2_O_2_ (100 μM) at different pH (5~9) values were studied (Figure 4b and Figure S9). The fluorescence intensity of probe **BT-BO** was weak and almost remained unchanged at a pH of 5~9, indicating that probe **BT-BO** was stabilized in the range of pH 5~9. While the fluorescence intensity of probe **BT-BO** + H_2_O_2_ demonstrated a volcanic curve, the maximal intensity was at a pH of 7. Consideration that the pH in the physiological environment was 7.4 and that probe **BT-BO** showed an optimal fluorescence intensity at a pH of 7, probe **BT-BO** could potentially detect endogenous H_2_O_2_ in living cells. What is more, the effect of the detection temperature of probe **BT-BO** was also evaluated. As Figure 4c shows, the fluorescence intensities of probe **BT-BO** when adding H_2_O_2_ were less influenced in the range of 26~34 °C, which shows another advantage of its utilization in living systems. The response time of probe **BT-BO** after adding H_2_O_2_ was investigated by measuring its fluorescence emission intensity (Figure 4d). After adding a 100 μM H_2_O_2_ solution, the fluorescence intensity increased up to 40 min, and then reached a plateau at 60 min. According to the first-order slope of the linear fitting curve (Figure 4d inner), the apparent reaction rate was 83.689 min^−1^, suggesting that probe **BT-BO** possesses a fast response time to H_2_O_2_.

### 2.5. Cytotoxicity and Cellular Imaging

**Figure 5:**
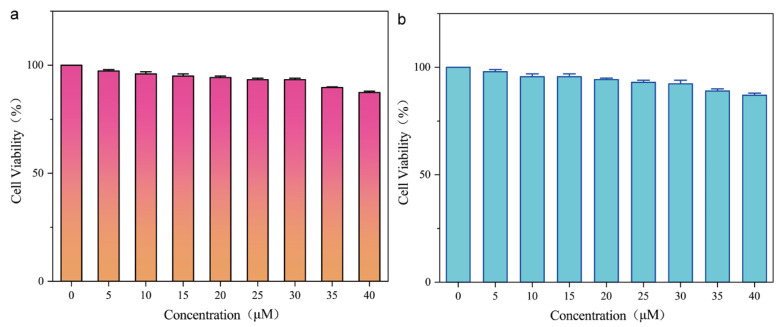
(**a**) A549 cells and (**b**) Hep G2 cells viabilities after being incubated with different concentrations (5, 10, 15, 20, 25, 30, 35 and 40 μM) of probe **BT-BO** for 4 h.

In order to investigate the probe’s performance in different cellular environments, the cytotoxicity and biocompatibility of probe **BT-BO** were evaluated by MTT assay using human pulmonary carcinoma A549 cells and human hepatocellular carcinoma Hep G2 cells. A series of different concentrations of **BT-BO** (0, 5, 10, 15, 20, 25, 30, 35 and 40 μM) were added to a cell culture medium. It can be clearly seen that there was no significant inhibition of cell growth with an increase in the **BT-BO** concentration for both A549 and Hep G2 cells (Figure 5a,b, respectively). The cell viability of A549 and Hep G2 cells measured by the MTT assay was over 94% and 91% at a **BT-BO** concentration of 30 μM, respectively, indicating that the designed probe possessed low-cytotoxicity cultured cells. Therefore, **BT-BO**, at a low concentration level (30 μM), was applied for the fluorescence imaging of A549 and Hep G2 cells. Furthermore, it was essential to explore the long-term stability of probe **BT-BO** in biological systems. Biological systems were simulated under a solution of 0.3% DMSO in PBS with probe **BT-BO** at 37 °C for 24 h. As shown in Figure S10, the purity of **BT-BO** (C_26_H_27_NO_3_SB, *m*/*z*: calcd. 444.1805 [M + H]^+^, found 444.160) was 99.7%. When probe **BT-BO** was dissolved in the simulated biological system (0.3% DMSO in PBS) at 37 °C for 24 h, the purity of **BT-BO** was slightly reduced, but still maintained at 95.2%. The above results indicate that the synthesized probe, **BT-BO**, possesses high purity and long-term stability in biological systems.

**Figure 6:**
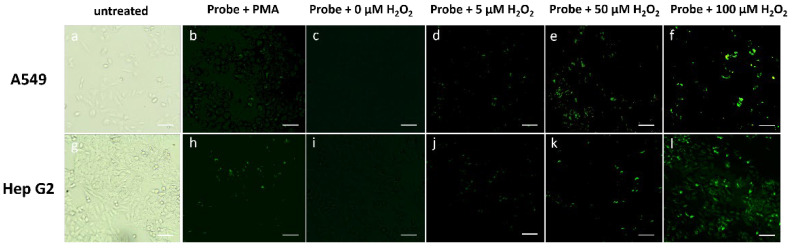
(**a**) Image of untreated A549 cells; (**b**) image of A549 cells incubated with probe BT-BO and PMA for 30 min; (**c**–**f**) image of A549 cells with exogenous H_2_O_2_ (0, 5, 50 and 100 μM, respectively) and probe BT-BO for 30 min; (**g**) image of untreated Hep G2 cells; (**h**) image of Hep G2 cells incubated with probe BT-BO and PMA for 30 min; (**i**–**l**) image of Hep G2 cells with exogenous H_2_O_2_ (0, 5, 50 and 100 μM, respectively) and probe BT-BO for 30 min. Scale bar = 50 μm.

Furthermore, to continue delving into the biological applications of probe **BT-BO**, two typical human cancer cells (A549 cells and Hep G2 cells) were used to study the applicability of **BT-BO** in living systems. As shown in Figure 6a,g, untreated A549 and Hep G2 cells both exhibit no fluorescence themselves. When the cells were cultured with **BT-BO** only, there was negligible fluorescence on their own (Figure 6c,i). However, after treating A549 and Hep G2 cells with PMA (a well-known kind of inducer for endogenous H_2_O_2_ generation) for 30 min and subsequently incubating them with probe **BT-BO**, a green fluorescence was dimly discernible and observed (Figure 6b,h, respectively); this suggested that endogenous H_2_O_2_ could be detected by probe **BT-BO**. To further investigate the detectability of probe **BT-BO** on A549 cells with exogenous H_2_O_2_, a range of H_2_O_2_ (5, 50, 100 μM) concentrations were introduced into A549 and Hep G2 cells (Figure 6d,e,f,j–l, respectively). Clearly, the fluorescence of both A549 and Hep G2 cells became stronger along with the increase in the H_2_O_2_ concentration. The above cellular imaging results indicate that probe **BT-BO** could detect both endogenous and exogenous H_2_O_2_ and could be a potential probe for the detection of H_2_O_2_ in living cells.

## 3. Materials and Methods

### 3.1. Materials and Measurements

2-hydroxybenzaldehyde, 2-aminobenzenethiol and 4-bromomethylphenylboronic acid pinacol ester were purchased from Accela ChemBio Co., Ltd. (Shanghai, China). 3-(4,5-dimethylthiazole-2)-2,5-diphenyltetrazolium bromide (MTT), dimethyl sulfoxide (DMSO) and formic acid were purchased from Shanghai Macklin Biochemical Technology Co., Ltd. (Shanghai, China). Anhydrous ethanol, anhydrous acetonitrile, potassium carbonate, hydrochloric acid and sodium hydroxide were purchased from TCI (Shanghai, China) Development Co., Ltd. Fetal bovine serum (FBS), Dulbecco’s modified Eagle’s medium (DMEM) and penicillin were purchased from Invitrogen Thermo Fisher Scientific—CN (Shanghai, China).

The ^1^H NMR and ^13^C NMR spectra were measured on a Bruker Avance III 400 MHz narrow-cavity liquid NMR spectrometer (Bruker, Bremen, Germany). Thin-layer chromatography (TLC) was carried out on a silica gel (GF254, Qingdao Haiyang Chemical Co., Ltd., Qingdao, China). The fluorescence spectra were obtained using a fluorescence spectrophotometer (F-2710, Hitachi, Hiroshima-shi, Japan) under 700 V, EX 2.5 nm and EM 2.5 nm.

### 3.2. Synthesis of the Fluorescence Probe BT-BO

#### 3.2.1. Synthesis of 2-(2’-Hydroxyphenyl)Benzothiazole (HBT)

The 2-hydroxybenzaldehyde (244.2 mg, 2 mmol), anhydrous ethanol (10 mL) and 2-aminobenzenethiol (275.4 mg, 2.2 mmol) were added to a round-bottom flask (50 mL) in sequence. Then, two drops of formic acid, to be used as a catalyst, were added to the solution above. The temperature of the mixed solution was raised to 90 °C, and the solution was heated at 90 °C to reflux for 2.5 h. The progress of the reaction was monitored using thin-layer chromatography (TLC). After the reaction was finished, the obtained precipitate was filtrated and washed with cold anhydrous ethanol 5 times and dried under an infrared lamp to give HBT (2-(2’-hydroxyphenyl)benzothiazole), a faint yellow product (350 mg, yield of 76.5%). ^1^ H NMR (400 MHz, DMSO-*d*_6_) δ 11.62 (1H, s, OH), 8.19 (1H, d, *J* = 8.0 Hz, ArH), 8.16 (1H, d, *J* = 8.0 Hz, ArH), 8.08 (1H, d, *J* = 8.0 Hz, ArH), 7.56 (1H, t, *J* = 8.0 Hz, ArH), 7.43–7.48 (2H, m, ArH), 7.11 (1H, d, *J* = 8.0 Hz, ArH), 7.04 (1H, t, *J* = 8.0 Hz, ArH). ^13^C NMR (100 Hz, DMSO-*d*_6_) δ 165.9, 156.8, 151.9, 134.6, 132.9, 129.0, 126.9, 125.6, 122.6, 122.4, 120.2, 118.7, 117.5. HR-MS: *m*/*z* (ESI^+^): C_13_H_10_NOS, calcd. 228.0483 [M + H]^+^, found 228.0487.

#### 3.2.2. Synthesis of 2-(2-((4-(4,4,5,5-Tetramethyl-1,3,2-Dioxaborolan-2-Yl)Benzyl)Oxy)Phenyl)Benzo[*d*]Thiazole (Probe BT-BO)

The HBT (229 mg, 1.0 mmol) was added into a flask of anhydrous acetonitrile (5 mL). After homogeneously mixing the solution, 4-(Bromomethyl) benzene boronic acid pinacol ester (356.4 mg, 1.2 mmol) and K_2_CO_3_ (165.6 mg, 1.2 mmol) were added into the solution above. The mixture was heated at 90 °C to reflux for 4 h and then cooled to room temperature. A yellow solid (probe **BT-BO,** (2-(2-((4-(4,4,5,5-tetramethyl-1,3,2-dioxaborolan-2-yl)benzyl)oxy)phenyl)benzo[*d*]thiazole, 339.9 mg, yield of 76.4%) was obtained by filtrating and purifying the solution above. ^1^ H NMR (400 MHz, CDCl_3_, TMS) δ 8.58 (1H, d, *J* = 8.0 Hz, ArH), 8.11 (1H, d, *J* = 8.0 Hz, ArH), 7.92 (1H, d, *J* = 8.0 Hz, ArH), 7.89 (2H, d, *J* = 8.0 Hz, ArH), 7.58 (2H, d, *J* = 8.0 Hz, ArH), 7.50 (1H, t, *J* = 8.0 Hz, ArH), 7.43 (1H, t, *J* = 8.0 Hz, ArH), 7.38 (1H, t, *J* = 8.0 Hz, ArH), 7.16 (1H, t, *J* = 8.0 Hz, ArH), 7.11 (1H, t, *J* = 8.0 Hz, ArH), 5.38 (2H, s, CH_2_), 1.35 (12H, s, CH_3_).^13^C NMR (100 Hz, DMSO-*d*_6_) δ 162.6, 156.3, 152.0, 140.0, 135.9, 135.1, 132.7, 129.5, 127.8, 126.7, 125.4, 122.9, 122.3, 122.0, 121.8, 114.3, 84.1, 70.6, 25.1. HR-MS: *m*/*z* (ESI^+^): C_26_H_27_NO_3_SB, calcd. 444.1805 [M + H]^+^, found 444.1801.

### 3.3. Liquid Chromatography–Mass Spectrometry

Experiments to determine the probe’s long-term stability in simulated biological systems were performed using a Waters high-performance liquid chromatography–tandem mass spectrometer (Milford, MA, USA) and quadrupole detector (model: liquid phase 2695–2996, mass spectrometry ZQ2000, Waters, Milford, MA, USA). The mobile phase comprised formic acid:water (1:100, vol:vol) and formic acid:acetonitrile (1:100, vol:vol). The liquid chromatography conditions used were as follows: Waters Sunfire C18 column (4.6 × 100 mm, 3.5 μm); column temperature, 30 °C; detection wavelengths, 214 nm and 254 nm; flow rate, 0.8 mL/min; and injection volume, 10 μL. The mass spectral conditions were as follows: electric spray ionization (ESI) source, negative ion ionization mode; capillary voltage, 3.0 kV; cone hole voltage, 10 V; source temperature, 140 °C; ionization temperature, 350 °C; ionization gas flow rate, 800 L/h; and cone hole gas flow rate, 50 L/h [47]. The detection molecular weight range was 360~560 *m*/*z*, and substances were quantitatively analyzed based on MS ES+ signals. Biological systems were simulated under a solution of 0.3% DMSO in PBS with probe **BT-BO** (10 μM) at 37 °C for 24 h.

### 3.4. Cytotoxic Assay

The cytotoxicity of probe **BT-BO** towards A549 and Hep G2 cells was investigated by the standard 3-(4,5-dimethylthiazole-2)-2,5-diphenyltetrazolium bromide (MTT) method. Typically, about 5 × 10^4^ A549 cells per well were seeded in 96-well plates for 24 h, and incubated with certain concentrations of probe **BT-BO** (0, 5, 10, 15, 20, 25, 30, 35 and 40 μM) at 37 °C for another 12 h. Then, 100 μL of the MTT solution was added into each well, followed by incubation under 5% CO_2_ at 37 °C in the dark for another 4 h. After this, the culture medium was removed and 100 μL of DMSO was added to dissolve the formed formazan. Each well was analyzed by the micro plate reader (Biotek Epoch, Winooski, VT, USA) at an absorbance of 490 nm. The cell viability was measured as a percentage of the control cells (blank) by taking the mean value of three replicate wells.

### 3.5. Cell Image Experiments

A549 and Hep G2 cells were chosen to study the living cell imaging. For a typical round, A549 and Hep G2 cells were cultured in DMEM on 35 mm dishes and incubated with penicillin (1%, vol/vol) and FBS (10%, vol/vol) at 37 °C in a 5% CO_2_ atmosphere for 24 h. All cell culture medium was removed and washed by PBS three times before the cell image experiments. For the endogenous H_2_O_2_ imaging experiment, A549 and Hep G2 cells were pretreated by using the activator PMA (phorbol-12-myristate-13-acetate, 1 µM) for 30 min and then treated with probe **BT-BO** (30 μM) for another 30 min. Subsequently, probe **BT-BO** was washed by PBS three times. For the detection of exogenous H_2_O_2_, A549 and Hep G2 cells were incubated with probe **BT-BO** (30 μM) for 30 min at 37 °C; the cells were then washed with PBS three times to remove excess probe **BT-BO**. Subsequently, H_2_O_2_ (0, 5, 50 and 100 μM) was introduced into cells for 30 min. Cell images were collected by an inverted fluorescence microscope (Nikon Ts2-FL, Tokyo, Japan) using blue bandpass filters with excitation wavelengths of 470 nm at 30 W, and a time frame of 10 ms~2 s. Images were captured at 40 × magnification (Table S4).

### 3.6. DFT Calculations

The structure of the compounds was optimized by using Chem3D Pro 14.0 to obtain the optimal structures. Then, theoretical calculations were performed using the B3LYP/6–31 + G method.

## 4. Conclusions

In summary, a novel “turn-on” fluorescent probe, namely 2-(2-((4-(4,4,5,5-tetramethyl-1,3,2-dioxaborolan-2-yl)benzyl)oxy)phenyl)benzo[*d*]thiazole (probe **BT-BO**), with high sensitivity to H_2_O_2_ in living cells was designed and synthesized successfully for the first time via a two-step simple chemical reaction with a high yield of 76.4% and purity of 99.7%. Probe **BT-BO** exhibited a well-resolved emission peak at 604 nm and the ability to prevent the interference of ROS, various metal ions, and anion ions. Moreover, the probe possessed the excellent properties of a broad pH range, a fast response time to H_2_O_2_ and low cytotoxicity. Finally, we successfully applied probe **BT-BO** to image A549 and Hep G2 cells to monitor both exogenous and endogenous H_2_O_2_. Overall, it is believed that the designed 2-(2-((4-(4,4,5,5-tetramethyl-1,3,2-dioxaborolan-2-yl)benzyl)oxy)phenyl)benzo[*d*]thiazole could be a potential probe for the diagnosis of H_2_O_2_-related diseases in living systems.

## Data Availability

Data are contained within the article and Supplementary Materials.

## Supplementary Materials

The following supporting information can be downloaded at: https://www.mdpi.com/article/10.3390/molecules29215181/s1, Figure S1, UV Absorbance spectra of **HBT**, **BT-BO**, **BT-BO** with H_2_O_2_ and ^1^H NMR titration spectra of **BT-BO** upon addition H_2_O_2_. Figure S2: ^1^H NMR spectra of HBT; Figure S3: ^13^C NMR spectra of HBT; Figure S4: ^1^H NMR spectra of probe **BT-BO**; Figure S5: ^13^C NMR spectra of probe **BT-BO**; Figure S6: HR-MS spectra of HBT; Figure S7: HR-MS spectra of probe **BT-BO**. Figure S8: Molecular structure of probe **BT-BO**. Table S1: **check CIF report of probe BT-BO**. Table S2: Detailed explanations of the ^1^H NMR spectra interpretation of probe **BT-BO**. Table S3: Detailed explanations of the ^13^C NMR spectra interpretation of probe **BT-BO**. Figure S9: Fluorescence emission of probe **BT-BO** at different pH values. Figure S10: LC-MS spectra of probe **BT-BO**. Table S4: Comparison of **BT-BO** with other reported fluorescence probe for H_2_O_2_ Table S5: The microscope set-up for the imaging experiments. References [5,8,18,35,36,37,38,39,40,41,42,43,44,45] are cited in the Supplementary Materials.
