# Supplementary material for: A Novel Benzothiazole-Based Fluorescent AIE Probe for the Detection of Hydrogen Peroxide in Living Cells
Source: Molecules. 2024 Nov 1;29(21):5181. doi: 10.3390/molecules29215181 (PMC11547549; doi:10.3390/molecules29215181)
Supplement: Supplementary file 1 [file molecules-29-05181-s001.zip › molecules-3279723-supplementary.pdf]

Supporting Information

A Novel Benzothiazole-Based Fluorescent AIE Probe for the Detection of Hydrogen Peroxide in Living Cells

1. Figure S1 UV absorbance spectra of **HBT**, **BT-BO**, **BT-BO** with H_2O_2 and ^1H NMR titration spectra of **BT-BO** upon addition H_2O_2

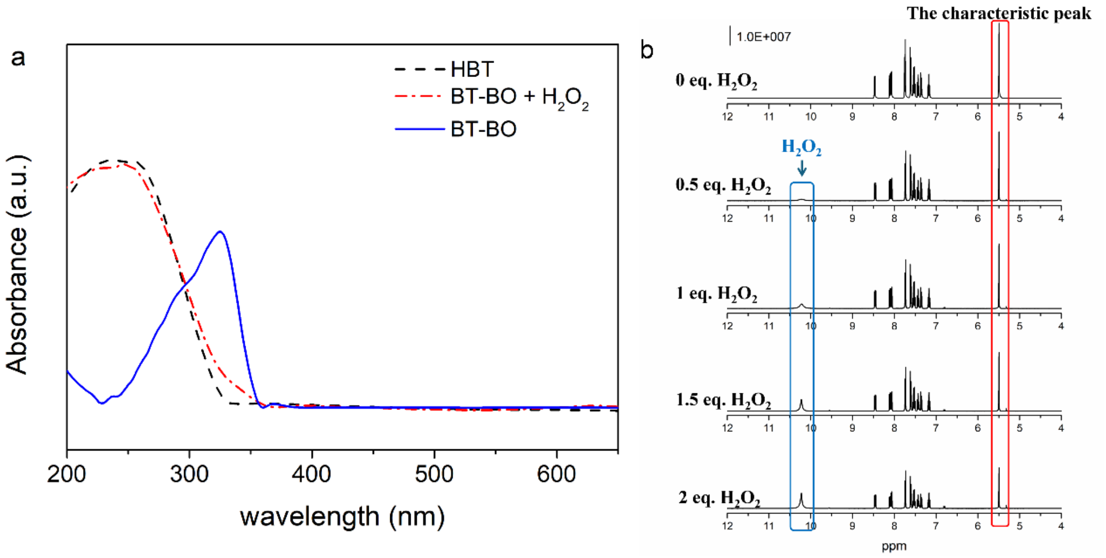

Figure S1. (**a**) UV Absorbance spectra of HBT (10 μM), BT-BO (10 μM) with H_2O_2 (200 μM) and BT-BO (10 μM); (**b**) ^1H NMR titration spectra of **BT-BO** upon addition of 0, 0.5, 1, 1.5 and 2 eq. H_2O_2

The aryl boric acid ester unit is a typical reactive group for H_2O_2. The possible reaction mechanism of aryl boric acid ester-based fluorescence probe involves the follow steps: first, the H_2O_2 attacks the boron atom of the borate moiety to remove a borate and release the negative ion of oxygen; and then through electron transfers and 1,6-eliminate of above obtained compound, the benzyl part is removed to obtain the fluorophore. In this work, the fluorophore (**HBT**) is attached to arylboronate ester moiety, therefore, probe **BT-BO** exhibits little fluorescence due to intramolecular interaction. When H_2O_2 reacts with the probe, arylboronate ester moiety is eliminated and the fluorophore is restored. By comparing with the UV-Vis absorption spectra of **HBT** and **BT-BO** + H_2O_2 (Figure S1a), obviously, the peak position and peak shape are striking resemblance for both of them, which provides one evidence for the possible reaction mechanism of aryl boric acid ester-based fluorescence probe, as mentioned in reported literature. To further confirm this possible reaction mechanism, ^1H NMR titration experiment was conducted to confirm the reaction mechanism. In the NMR chart (Figure S1b), δ(ppm) = 5.38 (methylene group, see Figure S4) is the is the characteristic peak regarding the recognition group [1]. After adding different equivalents of H_2O_2, the intensity of the characteristic peak gradually decreased, indicating that a chemical reaction occurred at the methylene group, which is in good agreement

with the UV absorbance spectra results. It is demonstrable that the recognition mechanism of this probe for hydrogen peroxide is logical.

2. Figure S2. ^{1}H NMR spectra of **HBT**.

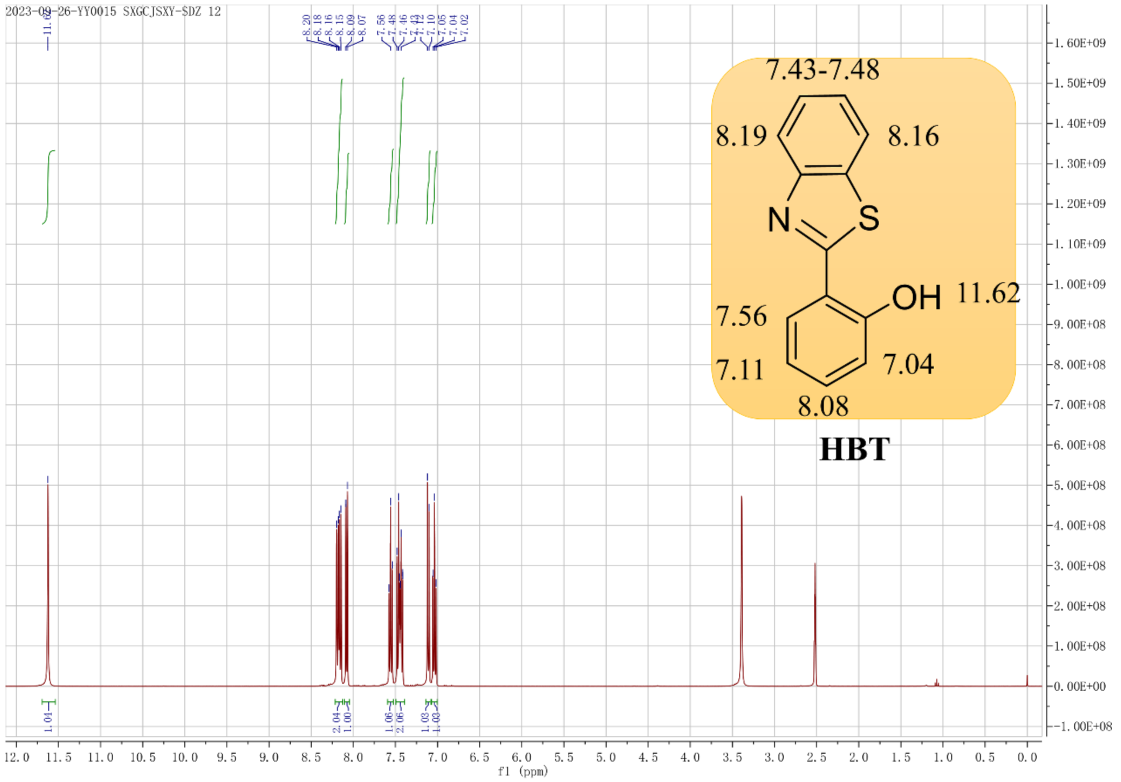

Figure S2. ^{1}H NMR spectra of HBT.

3. Figure S3. ^{13}C NMR spectra of HBT.

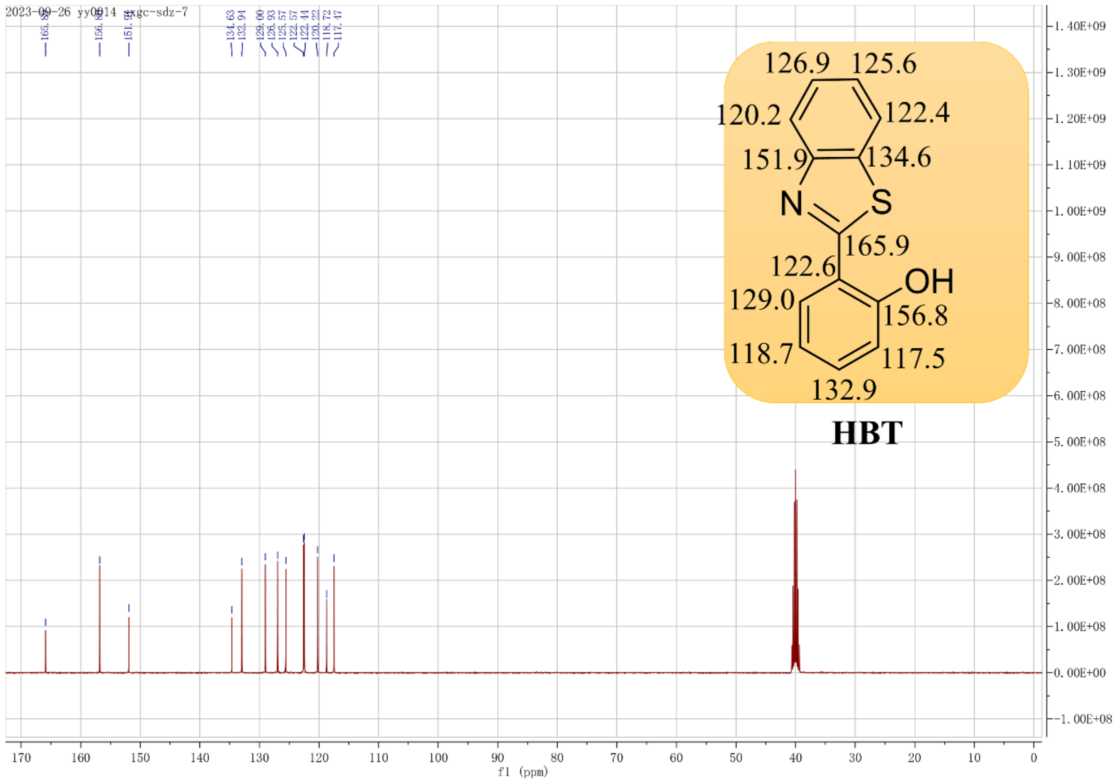

Figure S3. ^{13}C NMR spectra of HBT.

4. Figure S4. ^{1}H NMR spectra of probe **BT-BO**.

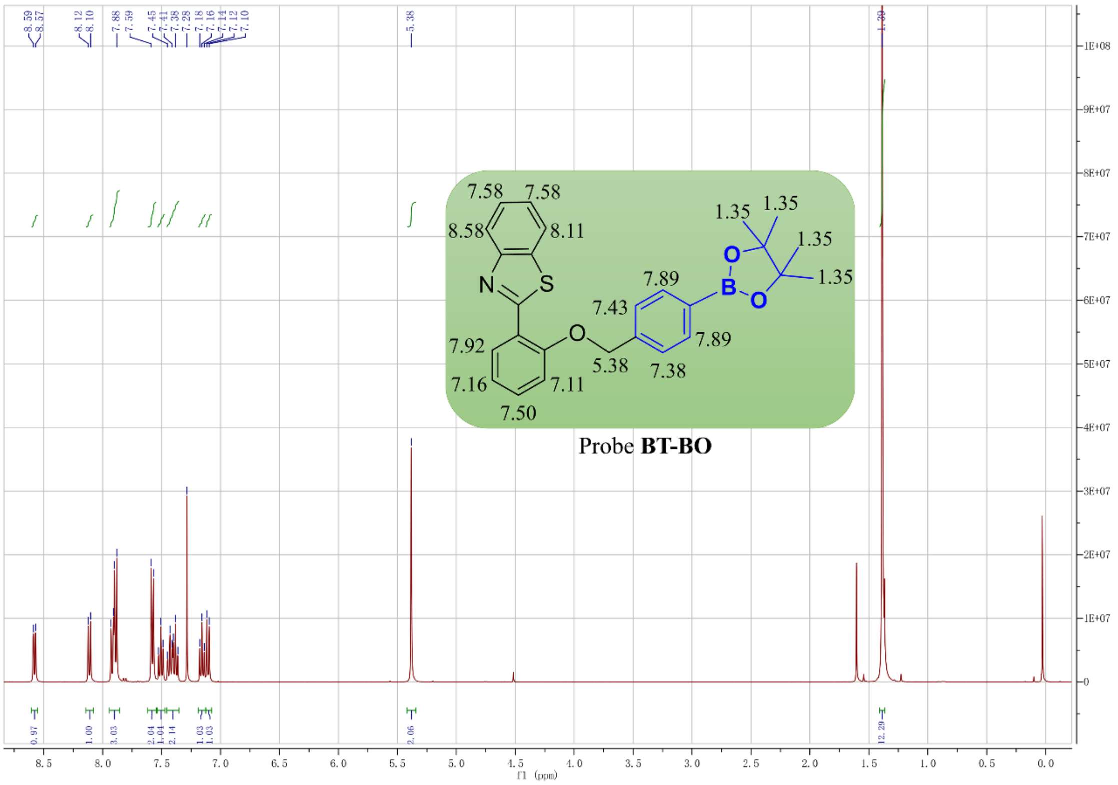

Figure S4. ^{1}H NMR spectra of probe **BT-BO**.

5. Figure S5. ^{13}C NMR spectra of probe **BT-BO**.

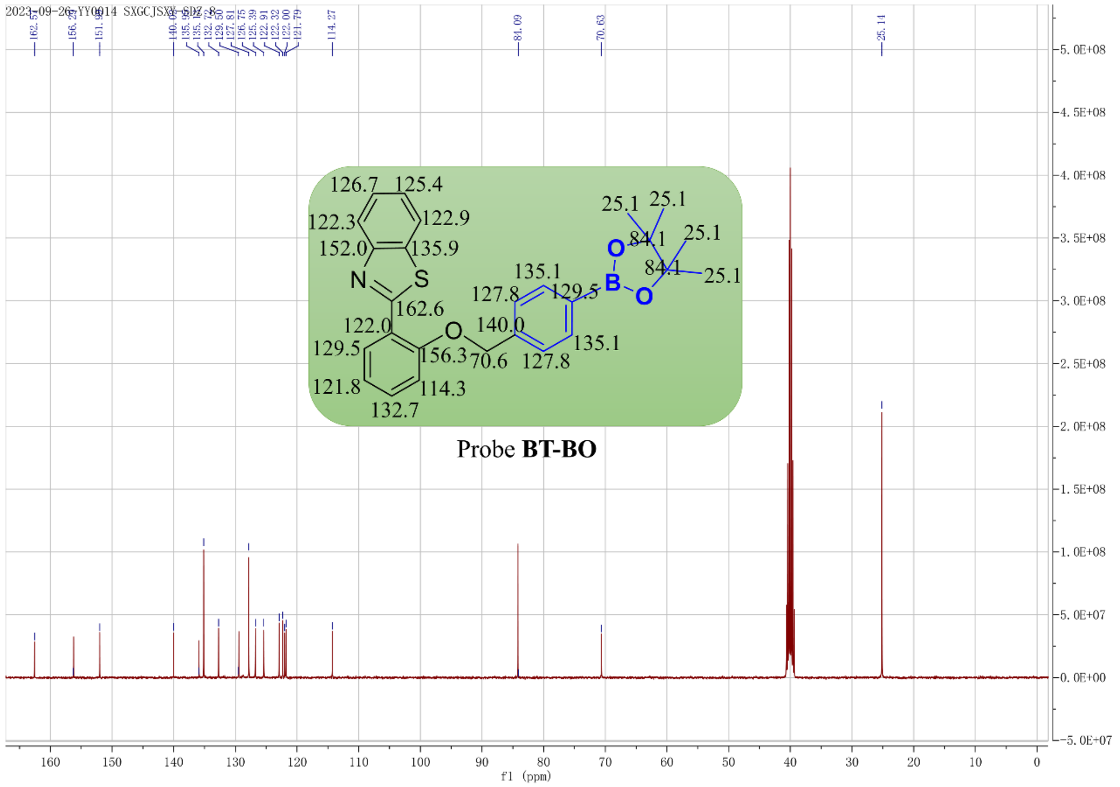

Figure S5. ^{13}C NMR spectra of probe **BT-BO**.

6. Figure S6. HR-MS spectra of HBT.

Elemental Composition Report **Page 1**

Single Mass Analysis
Tolerance = 5.0 mDa / DBE: min = -1.5, max = 50.0
Element prediction: Off
Number of isotope peaks used for i-FIT = 3

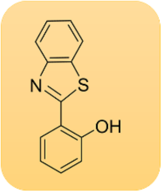

HBT

Monoisotopic Mass, Even Electron Ions
92 formula(e) evaluated with 1 results within limits (up to 50 best isotopic matches for each mass)
Elements Used:
C: 13-13 H: 10-10 N: 0-200 O: 0-200 S: 1-1
7
230927-13-439-1-7 18 (0.179)

1: TOF MS ES+
2.80e+004

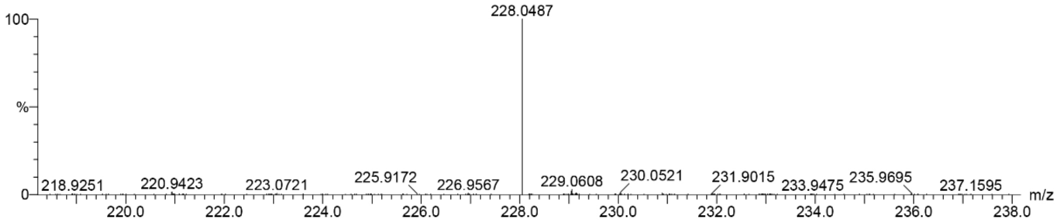

Minimum:				-1.5				
Maximum:		5.0	10.0	50.0				
Mass	Calc. Mass	mDa	PPM	DBE	i-FIT	Norm	Conf(%)	Formula
228.0487	228.0483	0.4	1.8	9.5	269.6	n/a	n/a	C13 H10 N O S

Figure S6. HR-MS spectra of HBT.

7. Figure S7. HR-MS spectra of probe **BT-BO**.

Elemental Composition Report Page 1

Single Mass Analysis
Tolerance = 5.0 mDa / DBE: min = -1.5, max = 50.0
Element prediction: Off
Number of isotope peaks used for i-FIT = 3

Monoisotopic Mass, Even Electron Ions
362 formula(e) evaluated with 1 results within limits (up to 50 best isotopic matches for each mass)
Elements Used:
C: 26-26 H: 27-27 N: 0-200 O: 0-200 S: 1-1 B: 1-1

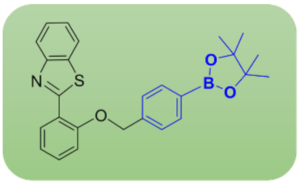

Probe **BT-BO**

7
230927-13-439-1-8 20 (0.205)

1: TOF MS ES+
4.37e+004

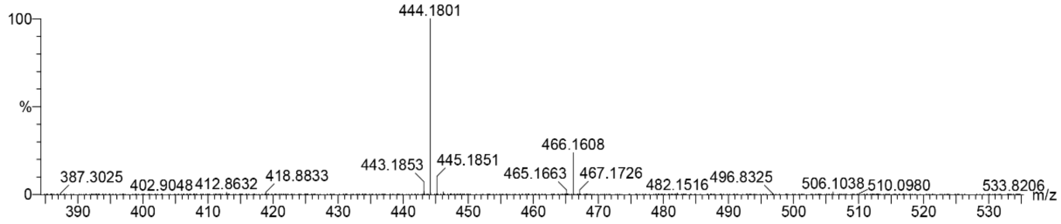

Minimum:				-1.5
Maximum:		5.0	10.0	50.0

Mass	Calc. Mass	mDa	PPM	DBE	i-FIT	Norm	Conf(%)	Formula
444.1801	444.1805	-0.4	-0.9	14.5	159.3	n/a	n/a	C26 H27 N O3 S B

Figure S7. HR-MS spectra of probe **BT-BO**.

8. Figure S8 Molecular structure of probe **BT-BO**

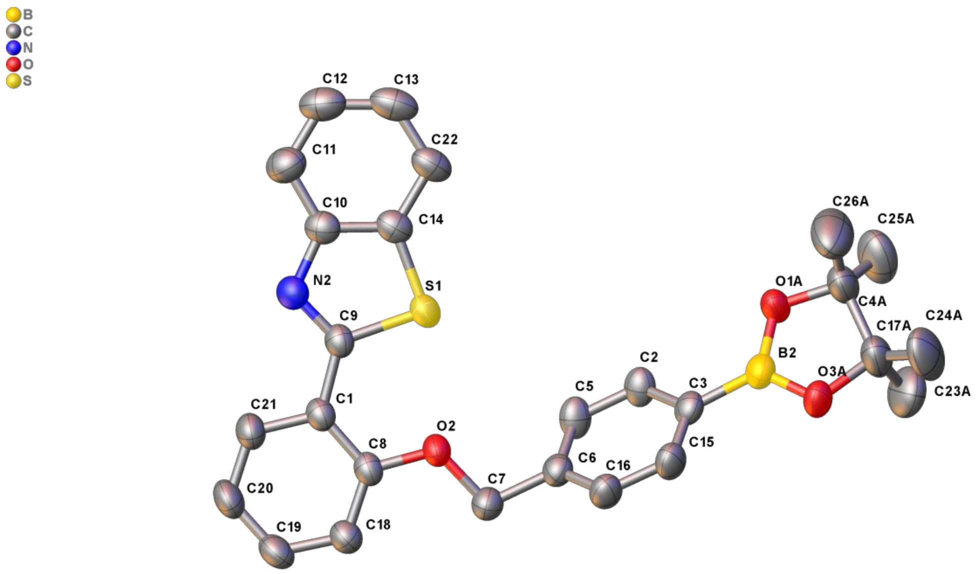

Figure S8 Molecular structure of probe **BT-BO**

9. Table S1 CheckCIF report of probe **BT-BO**

Cell	a (Å)	b (Å)	c (Å)
	6.7315(19)	15.001(4)	11.906(3)
	alpha (°)	beta (°)	gamma (°)
	90	91.048(12)	90
Items	Calculated	Reported	
Volume	1202.1(6) Å^3	1202.0(6) Å^3	
Space group	Pn	P1n1	
Hall group	P–2yac	P–2yac	
Moiety formula	C26H26BNO3S	C26H26BNO3S	
Sum formula	C26H26BNO3S	C26H26BNO3S	
Mr	443.35	443.35	
F000	468.0	468.0	
F000’	469.92		
h,k,l	8,18,14	8,18,14	

10. Table S2. Detailed explanations of the ^{1}H NMR spectra interpretation of probe **BT-BO**

**Node**	**Shift**	**Quantity**	**Comment**
ArH	8.58	1	aromatic H
ArH	8.11	1	aromatic H
ArH	7.92	1	aromatic H
ArH	7.89	2	aromatic H
ArH	7.58	2	aromatic H
ArH	7.51	1	aromatic H
ArH	7.43	1	aromatic H
ArH	7.38	1	aromatic H
ArH	7.16	1	aromatic H
ArH	7.11	1	aromatic H
CH_2	5.38	2	methylene
CH_3	1.35	12	methyl

11. Table S3. Detailed explanations of the ^{13}C NMR spectra interpretation of probe **BT-BO.**

**Node**	**Shift**	**Quantity**	**Comment**
CH	162.6	1	–C=N–
Ar–C	156.3	1	Ar–O
Ar–C-N	152.0	1	Ar–C–N=
Ar–C	140.0	1	Aromatic C
Ar–C	135.9	1	Ar–C–S
Ar–C	135.1	1	Aromatic C
Ar–C	132.7	1	Aromatic C
Ar–C	129.5	1	Aromatic C
Ar–C	127.8	1	Aromatic C
Ar–C	126.7	1	Aromatic C
Ar–C	125.4	1	Aromatic C
Ar–C	122.9	1	Aromatic C
Ar–C	122.3	1	Aromatic C
Ar–C	122.0	1	Aromatic C
Ar–C	121.8	1	Aromatic C
Ar–C	114.3	1	Aromatic C

C	84.1	2	aliphatic
CH_2	70.6	1	Aliphatic (Ar–C–O)
CH_3	25.1	12	aliphatic

12. Figure S9 Fluorescence emission of probe **BT-BO** at different pH values.

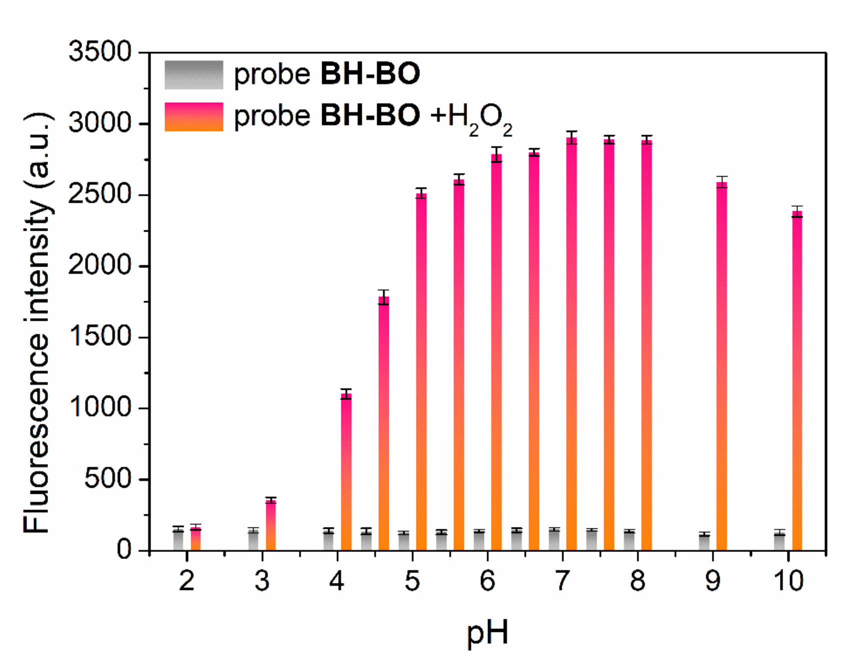

Figure S9. Fluorescence emission of probe **BT-BO** (5 μM) with (pink) or without (light gray) H_2O_2 at different pH values.

Considering the complex physiological environment in the organism, we examined the fluorescence response of the probe **BT-BO** over a wider pH range (from 2 to 10). The fluorescence intensity of probe **BT-BO** showed little changes in the absence of H_2O_2, indicating that the **BT-BO** is stable at various pH values. Nevertheless, the fluorescence intensity of **BT-BO** increased first and then decreased slightly with the increase of pH in the presence of H_2O_2. Obviously, the maximum fluorescence intensity value is around pH 7~7.5, which belongs to the physiological level of human body.

13. Figure S10. LC-MS spectra of probe **BT-BO.**

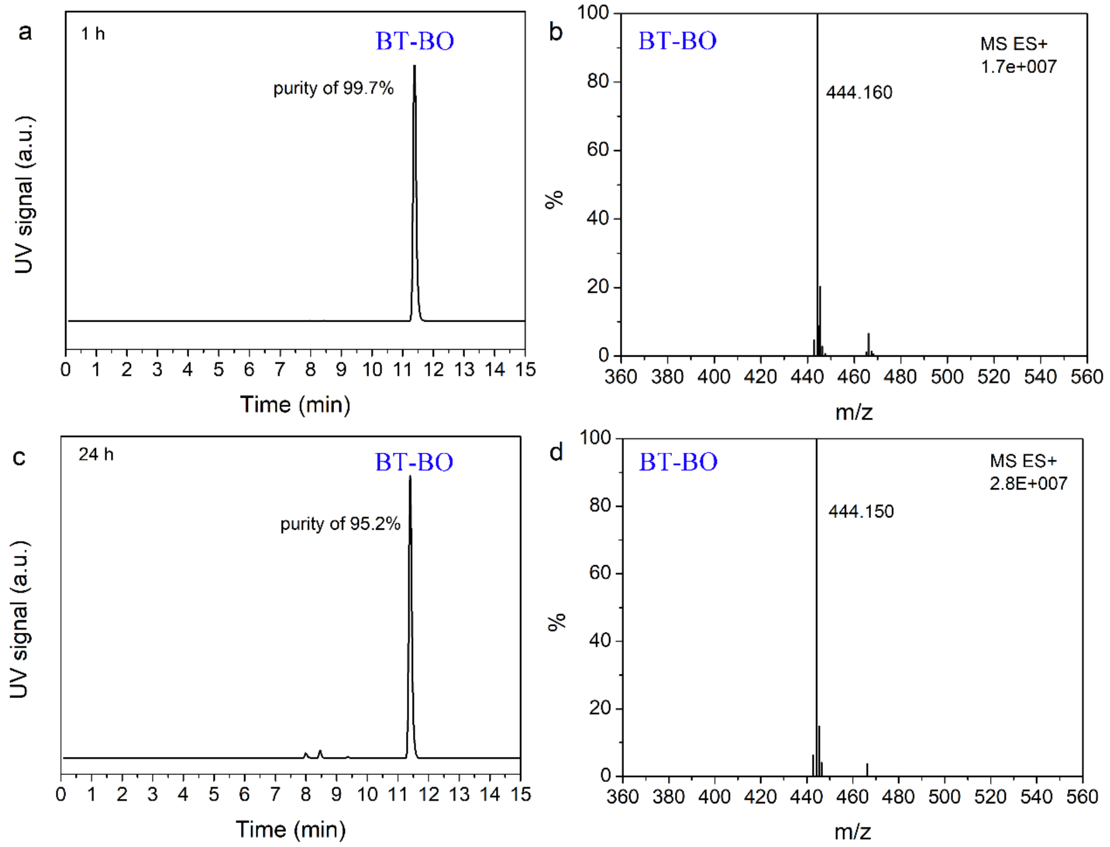

Figure S10. LCMS spectra of probe **BT-BO** in simulated biological system for 1 h (**a**) and 24 h (**c**). MS profile of probe **BT-BO** in simulated biological system for 1 h (**b**) and 24 h (**d**).

14. Table S4. Comparison of **BT-BO** with other reported fluorescence probe for H_2O_2

Probe	Limit of detection (LOD) (μM)	Response time (min)	Selectivity (-fold range over interferences)	Numbers of types of interferences	Application cells	Synthesis steps	References
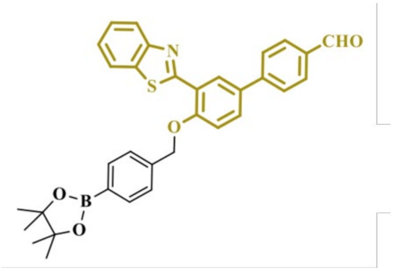	1.0	26	2.26~14.04	20	Hela	4 steps	[1]
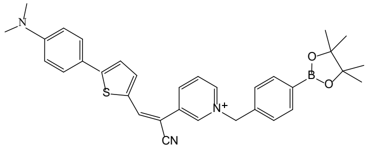	0.57	30	7.34~19.47	17	Raw 264.7<br>Hela	2 steps	[2]
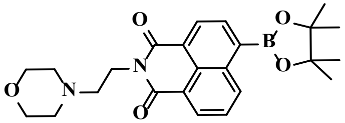	1.21	~2	13.75~34.37	13	HeLa<br>RAW 264.7	2 steps	[3]
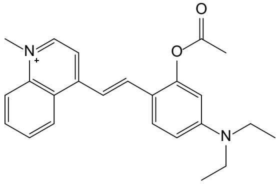	0.85	30	11.02~29.62	14	Hep G2	3 steps	[4]

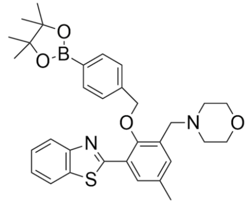	0.46	45	2.49~84.28	22	A549	4 steps	[5]
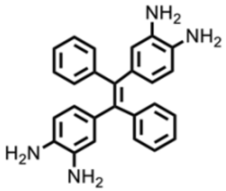	3.39	20	4.27~13.95	7	N.A.	2 steps	[6]
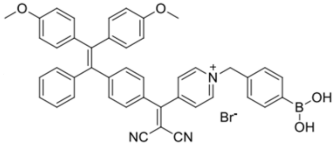	0.13	60	5.19~22.5	17	MCF-7	2 steps	[7]
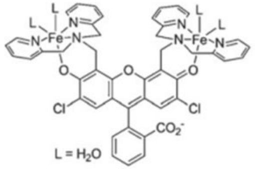	29	10	4.18~46	10	Hela<br>COS7	2 steps	[8]
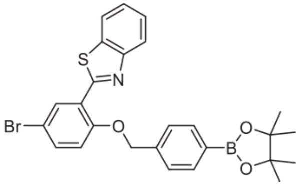	0.132	30	4.59~10.71	14	MCF-7	2 steps	[9]

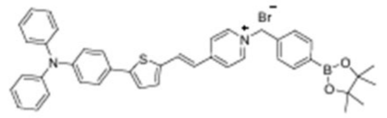	0.25	N.A.	N.A.	N.A.	HepG2<br>HeLa<br>Lo2	2 steps	[10]
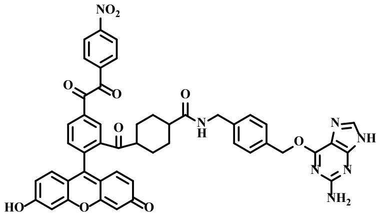	2.0	~7	N.A.	N.A.	RAW264.7	4 steps	[11]
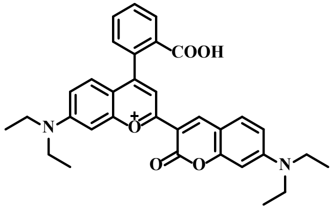	3.15	N.A.	9.26~19.23	15	RAW 264.7<br>HeLa	4 steps	[12]
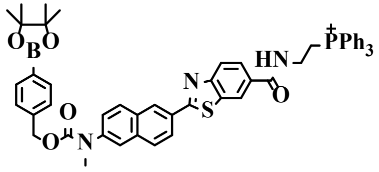	4.6	120	6.1~18.77	7	Raw 264.7	4 step	[13]
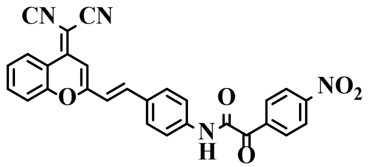	5.3	24	22.27~81.67	13	Hi-5	5 steps	[14]

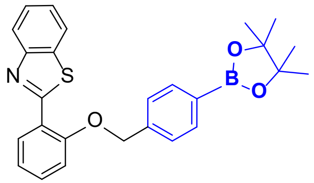	0.93	40	17.02~27.05	29	A549<br>Hep G2	2 steps	This work

15. Table S5: The microscope set-up for the imaging experiments

Model	Nikon Ts2-FL
Light filter used	Blue bandpass filters
Excitation wavelength	470 nm
Power	30 W
Time frame	10 ms - 2 s
Magnification	40 ×

References

[1] Tang, J.; Li, F.; Liu, C.; Shu, J.; Yue, J.; Xu, B.; Liu, X.; Zhang, K.; Jiang, W. Attractive benzothiazole-based fluorescence probe for the highly efficient detection of hydrogen peroxide. *Anal. Chim. Acta*, **2022**, *1214*, 339939.

[2] Liu, J.; Ji, L.; Yu, Y.; Rational design of a selective and sensitive "turn-on" fluorescent probe for monitoring and imaging hydrogen peroxide in living cells, *RSC Adv.*, **2021**, 11, 35093-35098.

[3] Ren, M.; Deng, B.; Wang, J. Y.; Kong, X.; Liu, Z. R.; Zhou, K.; He, L.; Lin, W.; A fast responsive two-photon fluorescent probe for imaging H_2O_2 in lysosomes with a large turn-on fluorescence signal, *Biosens. Bioelectron*, **2016**, *79*, 237-243.

[4] Han, H.; He, X.; Wu, M.; Huang, Y.; Zhao, L.; Xu, L.; Ma, P.; Sun, Y.; Song, D.; Wang, X. A novel colorimetric and near-infrared fluorescence probe for detecting and imaging exogenous and endogenous hydrogen peroxide in living cells, *Talanta*, **2020**, *217*, 121000.

[5] Zhu, N.; Xu, J.; Ma, Q.; Mao, G.; Zhang, J.; Li, L.; Liu, S. A new lysosome-targeted fluorescent probe for hydrogen peroxide based on a benzothiazole derivative. *Methods*, **2023**, *215*, 38-45.

[6] Li, X.; Yu, W.; Zhao, H.; Fan, Z.; Xiao, M.; Xi, R.; Xu, Y.; Meng, M.; Fluorogenic Biosensors Constructed *via* Aggregation-induced Emission Based on Enzyme-catalyzed Coupling Reactions for Detection of Hydrogen Peroxide, *Anal. Sci.*, **2021**, 37, 1275-1279.

[7] Jiang, G.; Li, C.; Liu, X.; Chen, Q.; Li, X.; Gu, X.; Zhang, P.; Lai, Q.; Wang, J.; Lipid Droplet-Targetable Fluorescence Guided Photodynamic Therapy of Cancer Cells with an Activatable AIE-Active Fluorescent Probe for Hydrogen Peroxide. *Adv. Optical Mater.,* **2020***, 8,* 2001119.

[8] Song, D.; Lim, J. M.; Cho, S.; Park, S. J.; Cho, J.; Kang, D.; Rhee, S. G.; You, Y.; Nam, W.; A fluorescence turn-on H_2O_2 probe exhibits lysosome-localized

fluorescence signals, *Chem. Commun.*, **2012**, *48*, 5449–5451.

[9] Ma, T.; Zhang, Y.; Fu, K.; Li, Z.; Yuan, C.; Ma, W.; Design, synthesis and properties of hydrogen peroxide fluorescent probe based on benzothiazole, *Bioorg. Chem.*, **2022**, *123*, 105798.

[10] Wu, Q.; Li, Y.; Li, Y.; Wang, D.; Tang, B. Z.; Hydrogen peroxide-responsive AIE probe for imaging-guided organelle targeting and photodynamic cancer cell ablation, *Mater. Chem. Front.*, **2021**, *5*, 3489-3496.

[11] Abo, M.; Minakami, R.; Miyano, K.; Kamiya, M.; Nagano, T.; Urano, Y.; Sumimoto, H.; Visualization of Phagosomal Hydrogen Peroxide Production by a Novel Fluorescent Probe That Is Localized via SNAP-tag Labeling, *Anal. Chem.*, **2014**, *86*, 5983-5990.

[12] Dong, B.; Song, X.; Kong, X.; Wang, C.; Tang, Y.; Liu, Y.; Lin, W.; Simultaneous Near-Infrared and Two-Photon In Vivo Imaging of H2O2 Using a Ratiometric Fluorescent Probe based on the Unique Oxidative Rearrangement of Oxonium, *Adv. Mater.* **2016**, DOI: 10.1002/adma.201602939.

[13] Masanta, G.; Heo, C. H.; Lim, C. S.; Bae, S. K.; Cho, B. R.; Kim, H. M.; A mitochondria-localized two-photon fluorescent probe for ratiometric imaging of hydrogen peroxide in live tissue, *Chem. Commun.*, **2012**, *48*, 3518–3520.

[14] He, Y.; Miao, L.; Yu, L.; Chen, Q.; Qiao, Y.; Zhang, J. F.; Zhou, Y.; A near-infrared fluorescent probe for detection of exogenous and endogenous hydrogen peroxide in vivo, *Dyes Pigments*, **2019**, *168*, 160-165.
